# Quantum Chemical Calculations on CHOP Derivatives—Spanning the Chemical Space of Phosphinidenes, Phosphaketenes, Oxaphosphirenes, and COP^−^ Isomers

**DOI:** 10.3390/molecules23123341

**Published:** 2018-12-17

**Authors:** Alicia Rey, Arturo Espinosa Ferao, Rainer Streubel

**Affiliations:** 1 Department of Organic Chemistry, Faculty of Chemistry, University of Murcia, Campus de Espinardo, 30100 Murcia, Spain; aliciareyplanells@gmail.com; 2 Institut of Inorganic Chemistry, Rheinischen Friedrich-Wilhelms-Universiy of Bonn, Gerhard-Domagk-Str. 1, 53121 Bonn, Germany

**Keywords:** main group elements, phosphinidene, phosphaketene, oxaphosphirene, phosphacyanic acid, phosphafulminic acid, oxaphosphirenide, phosphaethynolate

## Abstract

After many decades of intense research in low-coordinate phosphorus chemistry, the advent of Na[OCP] brought new stimuli to the field of CHOP isomers and derivatives thereof. The present theoretical study at the CCSD(T)/def2-TZVPP level describes the chemical space of CHOP isomers in terms of structures and potential energy surfaces, using oxaphosphirene as the starting point, but also covering substituted derivatives and COP^−^ isomers. Bonding properties of the P–C, P–O, and C–O bonds in all neutral and anionic isomeric species are discussed on the basis of theoretical calculations using various bond strengths descriptors such as WBI and MBO, but also the Lagrangian kinetic energy density per electron as well as relaxed force constants. Ring strain energies of the superstrained 1*H*-oxaphosphirene and its barely strained oxaphosphirane-3-ylidene isomer were comparatively evaluated with homodesmotic and hyperhomodesmotic reactions. Furthermore, first time calculation of the ring strain energy of an anionic ring is described for the case of oxaphosphirenide.

## 1. Introduction

**Scheme 1:**
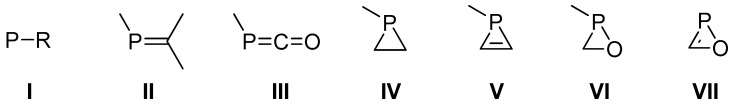
Low-coordinate (**I**–**III**) and strained (**IV**–**VII**) phosphorus compounds.

The chemistry of low-coordinate phosphorus compounds such as phosphinidenes (**I**), phosphaalkenes (**II**), and phosphaketenes (**III**) (Scheme 1) have received attention from experimentalists and theoreticians over many decades due to their particular bonding situation and reactivity [1,2,3,4]. Somewhat similar is the situation for strained ring systems containing phosphorus for which phosphiranes (**IV**) and 1*H*-phosphirenes (**V**) [5] may serve as good cases in point. An existing synthetic challenge is represented by oxaphosphiranes (**VI**) [6,7], only experimentally described as a ligand in transition metal complexes, being valuable due to a high potential in polymer chemistry, and oxaphosphirenes (**VII**) [8,9] (a 3-phosphinyl-1*H*-oxaphosphirene was computed as one of the possible CHOP isomers [10]) for which not even a (failed) attempt exists in the literature; the latter may stem from a (discouraging) degree of antiaromaticity [11]. The first stable derivative of a phosphaketene, possessing the general formula RP=C=O, was reported by Appel (R = Mes* = 2,4,6-tri-*tert*-butylphenyl) [12,13], and which remained the only derivative for a long time. It was Grützmacher who then described a novel approach to **III** using sodium phosphaethynolate Na[OCP] [14]. This new salt, bearing the OCP^−^ (phosphaethynolate) anion [15,16], enabled a systematic exploration of its use in the field of P-heterocyclic chemistry [17]. Interestingly, the anion reacts as an ambident nucleophile, i.e., it could be used to form transient phosphaalkynes that underwent cyclotrimerization to yield 1,3,5-triphosphinines [18,19] or to obtain new phosphaketene derivatives [20]. The parent phosphaketene (phosphaisocyanic acid) H–P=C=O has been recently isolated and spectroscopically characterized [21]. Both H–P=C=O and the PCO radical were prepared by reaction of CO with P atoms or PH radicals, which in turn were produced from P_4_ and PH_3_ discharge or photolysis [22]. Preliminary calculations on the geometry and relative energies of H–P=C=O [23,24] and its phosphacyanic acid (H–O–C≡P) isomer [25,26] have been reported, including their interconversion [27,28] and the less stable phosphafulminic acid (H–CPO) (at the MP2/DZ+P//HF/3-21G* level [28,29]). Worth mentioning is also a more recent study on compliance constants (reciprocals of the bond relaxed force constants) for the parent phosphaketene **III** and its “cyclic isomer H–P(μ-CO)” (1*H*-oxaphosphirene) **VII** [30].

**Figure 1:**
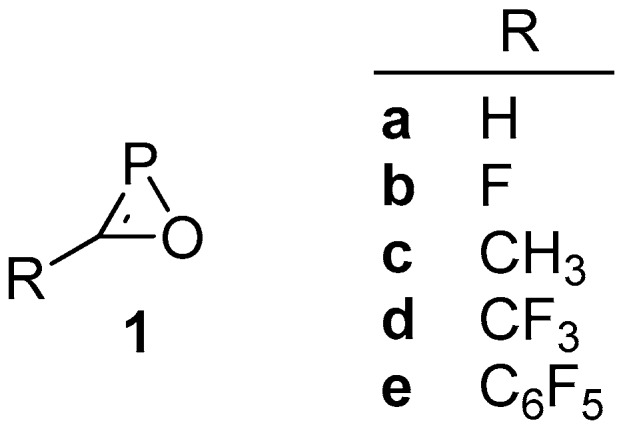
1*H*-Oxaphosphirene derivatives studied.

Herein, the potential energy surface (PES) for the parent 1*H*-oxaphosphirene (**1a**; hereafter, just oxaphosphirene) and four other substituted derivatives (**1b**–**e**) (Figure 1) were explored, constituting a representative set that covers electron-donating and -withdrawing groups. The PES for anionic derivatives of the formula COP^−^, conjugated base of **1a** and its isomers, is also presented. The ring strain energy for parent cyclic isomers is also estimated.

## 2. Results and Discussion

### 2.1. PES of the Parent Oxaphosphirene *(**1a**)*

**Scheme 2:**
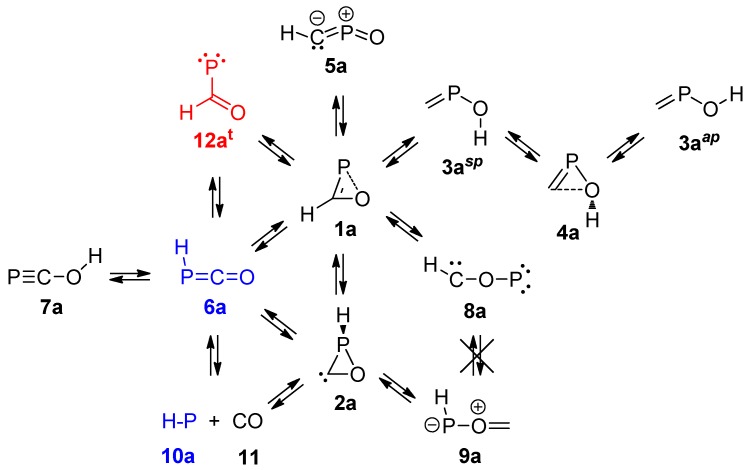
Isomers derived from parent oxaphosphirene **1a**. Singlet (closed-shell) structures represented in black, triplet ones in red, and those that were found in both electronic states are in blue.

The small ring size and the presence of a double bond in the oxaphosphirene ring in **1** is expected to entail high strain. Additionally, the availability of an electron pair at O together with the electron pair of the double bond could constitute an antiaromatic 4π electron arrangement that might be minimized by a geometric distortion. This energetically disfavourable combination should facilitate the existence of other (more) stable isomers, most of them being acyclic in nature. With this aim, the PES of the parent oxaphosphirene ring system **1a** was thoroughly explored and allowed the location of ten closed-shell molecular CHOP species, together with a fragmentation pathway (Scheme 2). Only significant differences with a previous report computed at the rather similar (see the Computational Details) QCISD(T)/6-311++G(3df,2p)//MP2/6-311++G(d,p) level [9] deserve comments. Another previous report described the geometry and relative energies of five of the closed-shell minima at the more modest B3LYP/6-311G** level [8].

First of all, in that report, the parent structure **1a** was described as an acyclic isomer having the connectivity of a formyl-phosphinidene (HC(O)–P) but featuring double C=P and single C–O bonds, as deduced exclusively from their bond distances (1.645 and 1.326 Å, respectively). Such a formyl-phosphinidene was claimed as minimum at the HF/6-31G** level (P–C 1.801 Å; C=O 1.193 Å; P–C–O angle 126.3°) [26], but never found at the working level of theory (see the Computational Details): the relatively small P–C–O bond angle of 86.5° found in **1a** (83.9°–88.7° for the full set of compounds **1**, except **1b** featuring an angle of 99.4°), far below the typical value for an open-chain C-sp^2^-centered bond angle (*ca.* 120°), strongly suggests the cyclic nature of **1a**, although with a certainly elongated endocyclic P–O bond (Table 1) for which no bond critical point (BCP) was found. Moreover, the “acyclic” structure “**3**” reported by Fu and coworkers [9] turns out to be erroneously drawn because, according to their data, the P–C–O angle (84.35°) is even more acute than the one reported herein for **1a** and, therefore, unambiguously corresponds to an oxaphosphirene species. The P–O bond in **1a** is by far the weakest (most flexible) bond according to its lowest relaxed force constant (*k*^0^ = 0.869 mdyn/Å), compared to the P–C (4.527 mdyn/Å) and C–O (5.510 mdyn/Å) bonds, in agreement with the reported compliance constants computed at the CCSD(T)(fc)/cc-pVTZ level [22]. The relaxed force constants *k*^0^, obtained from the compliance matrix method [31,32,33], have received much attention in recent years as a measure of bond strength in a variety of bonding situations including organophosphorus derivatives [30,34].

Lewis structural formulae for all closed-shell molecular isomers CHPO found **1a**–**9a** (Scheme 1) were drawn according to the best electronic description, as deduced from standard bond-strength descriptors, natural charges (Table 1), and NBO (natural bond orbital) analysis [35,36]. With this aim, widespread used bond orders quantities, such as the Wiberg bond index (WBI) [37] and the Mayer bond order (MBO) [38,39,40,41,42], were computed. They provide values approaching 1 for single bonds, 2 for double bonds, and so forth. Also, properties derived from the topological analysis of the electron density, in line with Bader’s AIM (atoms-in-molecules) theory [43,44,45], in particular the electron density itself (ρ) and the Lagrangian kinetic energy (*G*) computed at the bond critical point (BCP), were calculated. The latter was only recently used in characterizing bond strength in phosphorus-containing three-membered heterocycles [46] and, soon afterwards, the Lagrangian kinetic energy density per electron (*G*/ρ) was reported as a remarkable bond-strength property [47] which correlates with ring strain energies (within a series) when computed at the ring critical points [48].

**Figure 2:**
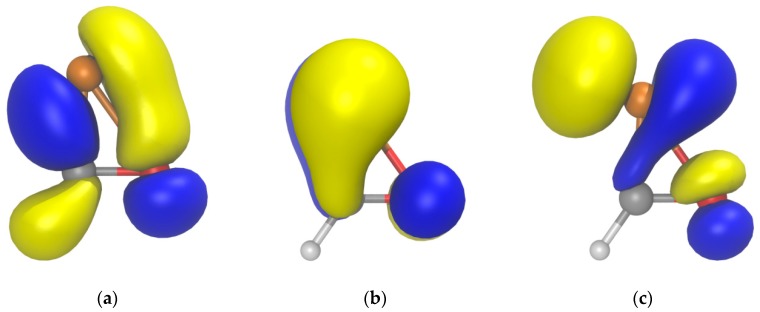
Computed (B3LYP/def2-TZVPP) Kohn-Sham isosurfaces (0.06 au) for (**a**) HOMO-1, (**b**) HOMO, and (**c**) LUMO of **1a**.

The rather weak P–O bond in **1a** can be attributed to two factors. On one hand, the rather ineffective single bond formed with π-symmetry between a p orbital at P and π*(C–O), as shown in the HOMO-1 (Figure 2a); the NBO analysis indicates the formation of the P–O single bond between two almost pure p orbitals at both P (98.31% p character) and O (98.15% p character). On the other hand, the antibonding π*(P–O) and σ*(P–O) character of the HOMO and LUMO (Figure 2b,c), respectively, the latter with a remarkable population (0.027 electrons) according to the NBO analysis.

The cyclic carbene, oxaphosphirane-3-ylidene **2a**, also displays a rather weak P–O bond (Table 1) but with higher stiffness (*k*^0^ = 1.358 mdyn/Å) compared to **1a**. In this case the C–O bond also varies in the same sense (*k*^0^ = 7.181 mdyn/Å), whereas the P–C bond becomes comparatively weaker and more elastic (*k*^0^ = 1.412 mdyn/Å).

**Scheme 3:**
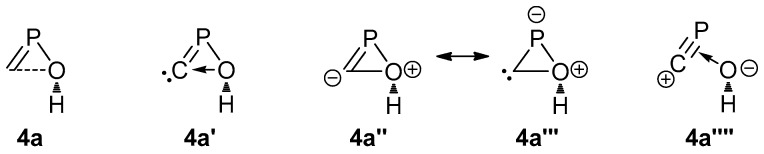
Different representations of isomer **4a**.

The large and moderately weak C–O bond (no BCP found) in **4a** (Table 1), suggests a dative bonding from an electron pair at O to a vacant orbital at the carbenic C atom (**4a′**), which is equivalent to the all-covalent bond Lewis formula **4a″** or its resonant structure **4a‴** (Scheme 3).

**Figure 3:**
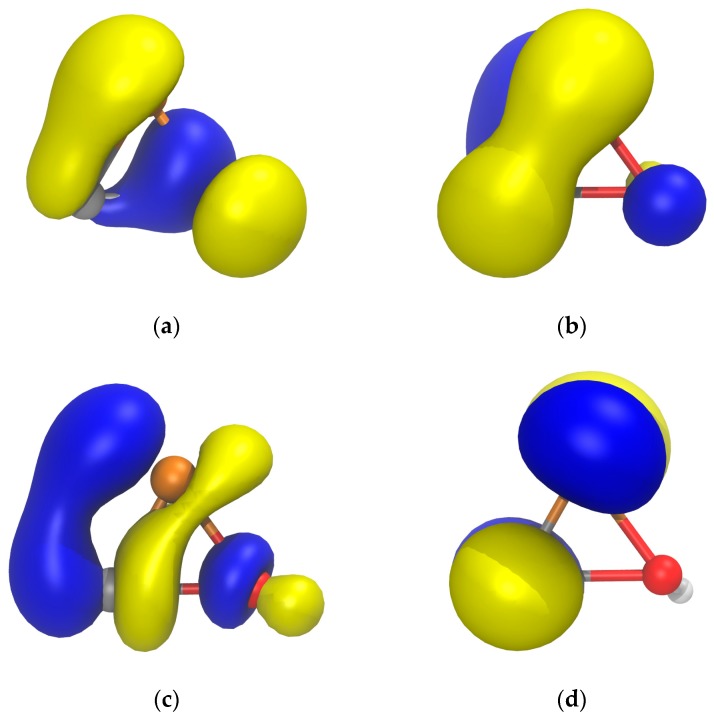
Computed (B3LYP/def2-TZVPP) Kohn–Sham isosurfaces (0.06 au) for (**a**) HOMO-3, (**b**) HOMO, (**c**) LUMO, and (**d**) LUMO+1 of **4a**.

The cyclic character of **4a** is supported by NBO analysis that shows a formal C–O single bond between two almost pure p orbitals at both C and O (96.63 and 93.74 % p character, respectively), the same holding for the single P–O bond (91.38 and 83.97% p character for P and O, respectively). This is compatible with a p-donor π-acceptor complex **4a⁗** between a hydroxide and a [C≡P]^+^ cation, according to the Dewar-Chatt-Duncanson model [49]. The MO drawings also support this view as far as HOMO-3 and LUMO constitute the bonding and antibonding interactions between an in-plane π(C=P) orbital with a p-type atomic orbital at O, whereas HOMO and LUMO+1 are essentially the π(C=P) and π*(C=P) combinations in the orthogonal plane (Figure 3).

It is worth mentioning that isomer **9a**, resulting from P–C bond cleavage in oxaphosphirene **1a** (Scheme 2), might be considered as a van der Waals complex between carbon monoxide (**11**) and singlet phosphinidene (**10a**) (C≡O→P–H), as deduced from the rather elongated and weak P–O linkage (Table 1). This bond might easily cleave, although the corresponding TS could not be located at the working level of theory and, hence, more information is not available.

**Figure 4:**
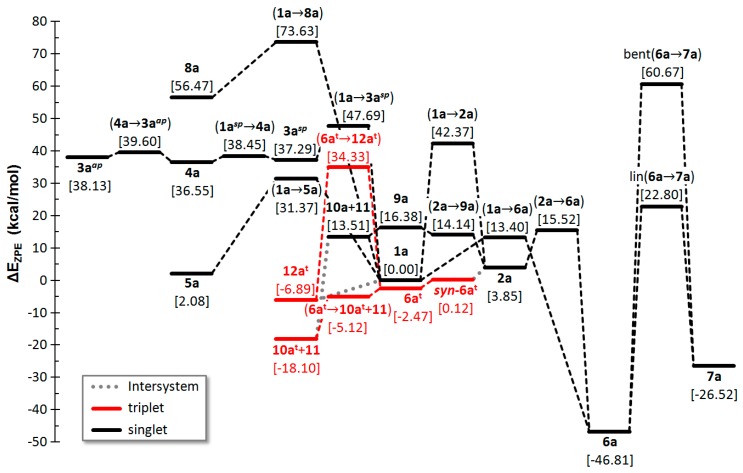
Computed (CCSD(T)/def2-TZVPP//B3LYP-D3/def2-TZVP) zero-point corrected energy profile for the interconversion of CHOP isomers.

All closed-shell minima are the same as in Fu’s report, with the only exception of isomer **3a*^ap^*** that was described with a linear POC moiety but, at the current level of theory, has a zig-zag geometry. Indeed, we have confirmed the linear arrangement upon geometry optimization at the SCS-MP2/def2-TZVP level. Species **3a*^ap^*** can only be formed from its most stable conformer **3a*^sp^*** through the cyclic isomer **4a**. The TS (transition state) for converting **3a*^sp^*** into **4a** is the only one not reported in Fu’s work. All ground states and TSs have similar energies at both computational levels (rmsd = 1.77 kcal/mol). A full energy diagram with all CHPO isomers and the interconversion paths is collected in Figure 4. The TS for the dissociation of **9a** into singlet phosphinidene (**10a**) and CO (**11**) could not be located.

The overall PES points to cyclic systems **1a** and **2a** as thermodynamically disfavoured compared to the most stable acyclic phosphaketene **6a** (and phosphacyanic acid **7a**) and displaying little kinetic stability, as deduced by the little barriers for their conversion into **6a**. The other cyclic isomer **4a** is rather unstable both thermodynamically and kinetically.

Although a formyl-phosphinidene was not located at the closed-shell PES of **1a**, excitation of **1a** to its first triplet state is accomplished with structural reorganization leading to the triplet formyl-phosphinidene **12a^t^** (Scheme 2, Figure 4), as previously recognized [8]. The same excitation in oxaphosphirane-3-ylidene **2a** leads to ***syn*-6a^t^** that isomerizes to the *anti* conformer **6a^t^**. The latter species can isomerize to the aforementioned species **12a^t^** or split into the “stable” species triplet phosphinidene (**10a^t^**) and carbon monoxide (**11**).

### 2.2. Isomers of Substituted Oxaphosphirenes

**Figure 5:**
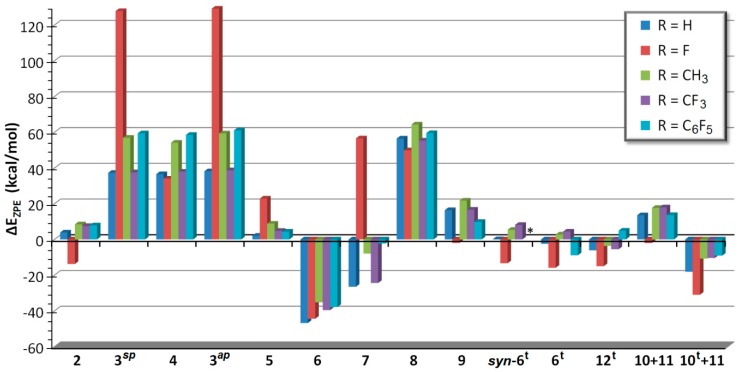
Computed (CCSD(T)/def2-TZVPP//B3LYP-D3/def2-TZVP) zero-point corrected relative energies for all isomers of oxaphosphirenes **1a**–**e**. Energies are referred to the oxaphosphirene species (**1**).

In case of the other differently substituted oxaphosphirenes **1a**–**e**, the energies of the same set of isomers was computed. In almost all cases the most stable closed-shell species follow the same order as for the parent compounds, starting by the most stable phosphaketene (phosphaisocyanate) R–P=C=O: **6** > **7** > **1** > **5** ≥ **2** > **9** (Figure 5). On the contrary, in case of the fluoro-substituted species, **7b** is strongly destabilized (also both conformers **3b** as well as **5b** to a lesser extent), whereas **2b** and **9b** (also **10b**) are comparatively stabilized. This results in a different order of stability **6b** > **2b** > **9b** > **1b** > **5b** > **7b** (Figure 4). Triplet electronic states display similar stability than (singlet) oxaphosphirenes **1**, with two general exceptions: 1) splitting into triplet phosphinidene (**10^t^**) and carbon monoxide (**11**) are usually favored and 2) the fluorinated substituent comparatively favors all triplet state species (***syn*-6b^t^**, **6b^t^** and **12b^t^**), especially the pair **10b^t^** + **11** (obtained after dissociation). Compound ***syn*-6e^t^** is not stable and spontaneously splits into **10e^t^** + **11**.

Therefore, the overall picture for the thermodynamic instability of cyclic isomers and the preference for phosphaketenes does not change significantly with the type of substitution (Figure 5), compared to the parent compounds (Figure 4), except a small relative stabilization of the oxaphosphirane-3-ylidene **2b**, and the remarkable destabilization of fluoro-phosphacyanate **7b**, in case of the fluoro-substitution.

### 2.3. Anionic COP^−^ Isomers

**Scheme 4:**
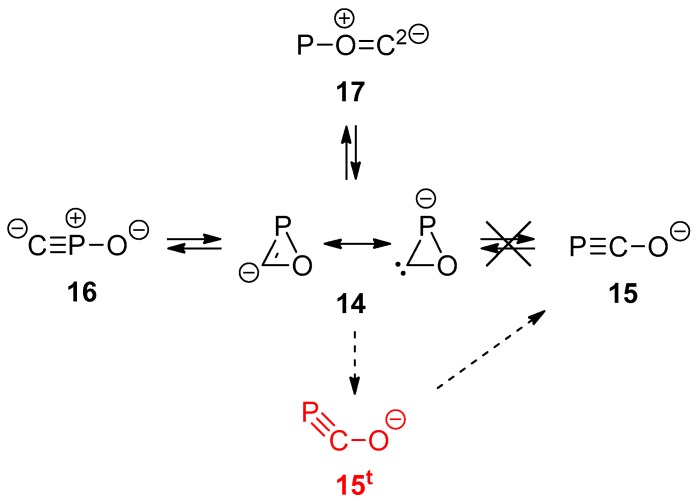
Isomers derived from oxaphosphirenide **14**. Singlet (closed-shell) structures represented in black and triplet ones in red.

**Figure 6:**
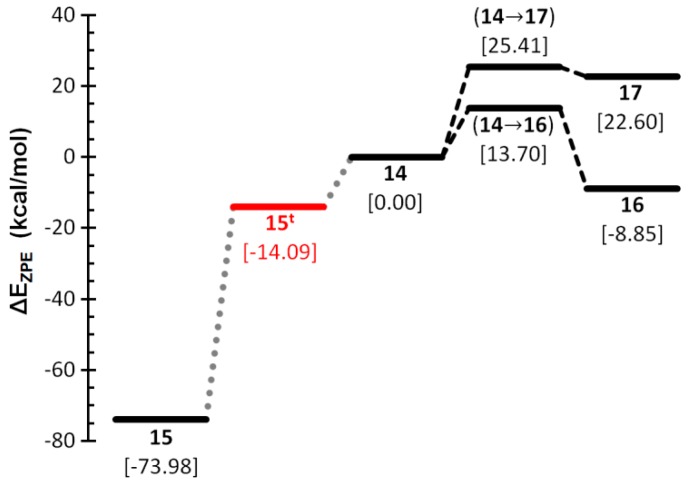
Computed (CCSD(T)/def2-TZVPP//B3LYP-D3/def2-TZVP) zero-point corrected energy profile for the interconversion of COP^−^ isomers.

Formal substitution of H in the parent compounds by a lone pair affords the set of anionic species of general formula CPO^−^, for which only four closed-shell minima were found: the oxaphosphirenide **14** and the products resulting from endocyclic P–O (**15**), C–O (**16**), and P–C (**17**) bond cleavage (Scheme 4). For only the two latter ring cleavage processes, the corresponding TSs were characterized. With a small barrier, the C–O bond cleavage of **14** affords exergonically CPO^−^ **16**, whereas cleavage of the P–C bond occurs through a moderately higher barrier and giving rise to the less stable isomer POC^−^ **17** (Figure 6). On the contrary, cleavage of the P–O bond under the restricted (closed-shell) formalism leads to splitting into CO and a P- anion, but excitation of **14** to the first triplet electronic state leads to a spontaneously cleavage of the P–O bond, affording the *bent* triplet species **15^t^** which, on falling to the electronic ground (singlet) state, enables the access to the most stable isomer, the *linear* phosphaethynolate anion PCO^−^ **15**.

**Figure 7:**
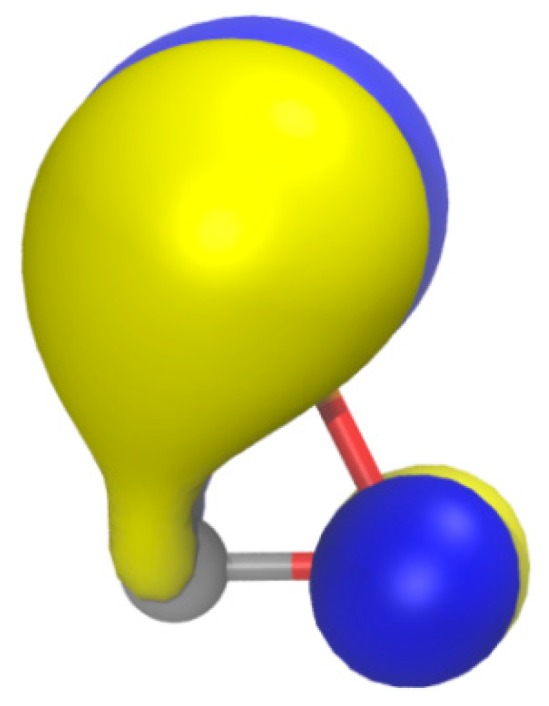
Computed (B3LYP/def2-TZVPP) Kohn-Sham isosurface (0.06 au) for the HOMO of **14**.

The oxaphosphirenide **14** can be formally considered as the direct deprotonation product from either **1a** (C-deprotonation) or **2a** (P-deprotonation). Although most of the negative (natural) charge can be ascribed to the O atom due to its higher electronegativity (Table 1), the largest negative variation in electric charge corresponds to the P atom (Δq^nat^ = −0.718 or −0.608 *e* in **1a** or **2a**, respectively) and, therefore, **14** could be safely represented with a formal negative charge on phosphorus. This is also supported by the HOMO (Figure 7) being mainly located at P. The stiffness of all three bonds keep the same order found for **1a** and **2a**: C–O > P–C > P–O (*k*^0^ = 4.322, 1.846 and 1.099 mdyn/Å, respectively).

### 2.4. Ring Strain Energy

**Scheme 5:**
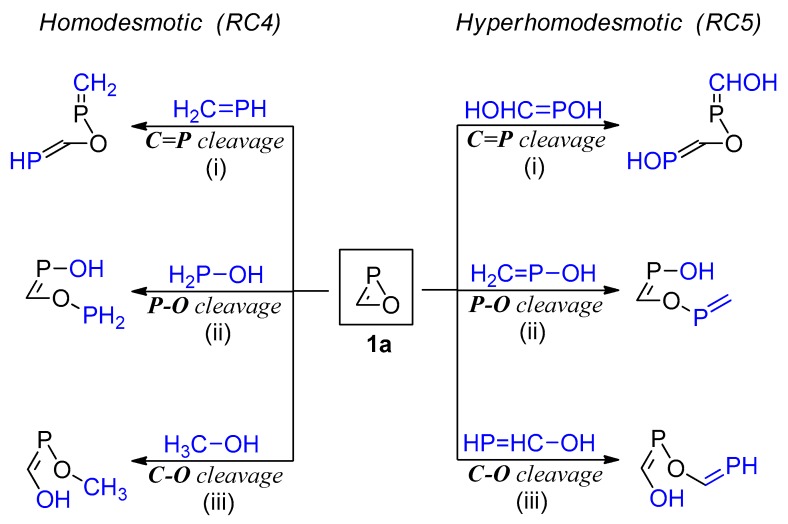
Homodesmotic and hyperhomodesmotic reactions used for the evaluation of the RSE in parent oxaphosphirene **1a**.

Ring strain is one of the most characteristic features in small rings, providing the driving force for their transformations into open-chain (or ring-enlarged) products [50]. The ring strain energy (RSE) for the parent saturated oxaphosphirane ring was reported to be 23.5 kcal/mol (computed at the CCSD(T)/def2-TZVPP level) [51]. As double bonds normally entail enlarged bond angles, their incorporation into small cyclic systems should be expected to result in an increase in the RSE, as occurs, for instance, in moving from cyclopropane to cyclopropene (RSE = 29.0 and 54.5 kcal/mol, respectively [52]). In the case of the parent oxaphosphirene ring system **1a**, the RSE was computed by evaluating the energetics of appropriate homodesmotic reactions (Scheme 5), similar to those used for other three- [46,48,51,53,54,55,56,57] or four-membered [47] saturated phosphorus heterocycles. In such reactions, for the cleavage of every endocyclic X–Y (or X=Y) bond, a H_n_X–YH_m_ (or H_n_X=YH_m_) reagent is used, the valences of X and Y being completed with H atoms. In reactants and products, the number of every type of bonds (single/double) between X and Y, with the same hybridization states in X and Y, must be conserved, as well as the number of sp^3^, sp^2^, and sp-hybridized (and non-hybridized, in case) atoms of every element with 0, 1, 2, or 3 H atoms attached. By averaging the opposites of the zero point-corrected energies for the three homodesmotic endocyclic bond cleavage reactions of **1a**, a rather high value of RSE (Table 2) was obtained (see Computational Details).

According to a recent classification and redefinition of reaction types used in thermochemistry [58], homodesmotic reactions (reaction class 4, or “RC4”) is the second to last type in a hierarchy of increasingly more accurate processes, due to conservation of larger fragments. In this hierarchy, hyperhomodesmotic reactions (reaction class 5, or “RC5”) constitute the top quality, most accurate type of processes. They are defined so as to conserve in reactants and products the same number of H_n_X–YH_m_ bonds (X, Y are the two bonded atoms; n and m are the number of H atoms attached to them), for every type of single, double, or triple bond, as well as the same number of sp^3^, sp^2^, and sp-hybridized (and non-hybridized, in case) atoms of every element with 0, 1, 2, or 3 H atoms attached. The small difference obtained in the RSE (Table 2) when using the RC4 and RC5 sets of reactions (*ca.* 0.8 kcal/mol) justifies the widespread use of homodesmotic reactions for the estimation of RSE without significant loss of accuracy. Moreover, the use of lower levels of theory resulted in an overestimation of RSE (RC4/RC5) for **1a** that increases from PWPB95-D3 (50.88/50.15 kcal/mol), B3LYP-D3 (52.05/51.19 kcal/mol) and SCS-MP2 (52.15/51.45 kcal/mol).

**Scheme 6:**
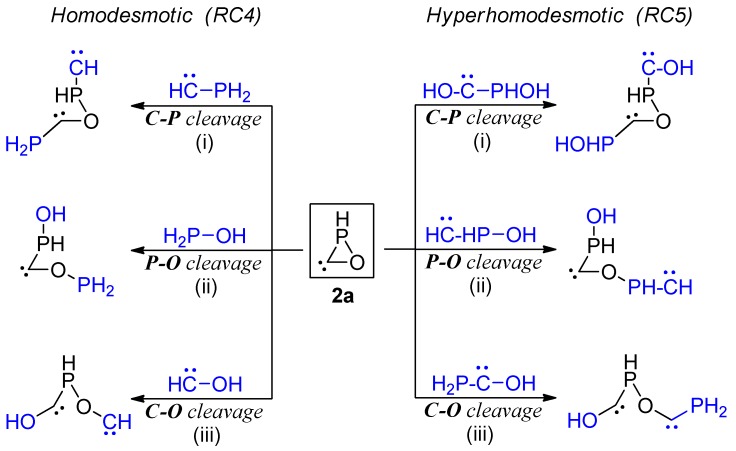
Homodesmotic and hyperhomodesmotic reactions used for the evaluation of the RSE in parent oxaphosphirane-3-ylidene **2a**.

The RSE for oxaphosphirane-3-ylidene **2a** was computed similarly by using the appropriate set of homodesmotic and hyperhomodesmotic reactions (Scheme 6). The results point to a very significant decrease in the RSE (more than 40 kcal/mol) compared to **1a**, most likely paralleling the decrease in bond order for the P–C linkage. This drop in RSE does not entail an overall stabilization of **2a** with respect to **1a** in the same magnitude, owing to the presence of very reactive (unstable) centers in **2a**, such as the carbene moiety.

**Scheme 7:**
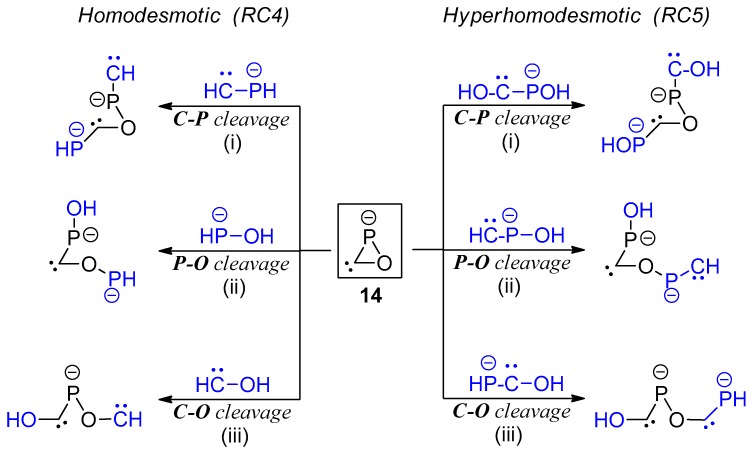
Homodesmotic and hyperhomodesmotic reactions used for the evaluation of the RSE in oxaphosphirenide anion **14**.

In case of the oxaphosphirenide **14**, the required use of anionic reagents for the appropriate homodesmotic and hyperhomodesmotic ring cleavage reactions (Scheme 7) forms cleavage products dianionic in nature. As the latter should display an extra Coulombic destabilization, not present in the (isolated) reagents, a compensation of the resulting repulsion energy was needed. This was estimated via the Coulomb law formalism. With this aim, the two non-consecutive atoms contributing the most to HOMO and HOMO-1 (or even HOMO-2 if the uppermost HOMOs are constrained to the same part of the molecule) are considered as centers for the two negative charges in the cleavage product. For this particular case, electrostatic repulsion energies in the range of 80 kcal/mol are obtained. The principal limitation of this methodology is that the use of point charges located at particular atom centers is nothing but a crude simplification of the real electron density distribution and, consequently, some minor source of inaccuracy must be assumed. Thus, by using only the homodesmotic C–O cleavage reaction (type iii RC4, Scheme 7) that results in a monoanionic acyclic product and therefore not needing any further Coulombic energy correction, an RSE estimation of 38.56 kcal/mol was obtained. After averaging with the other two Coulomb term-corrected homodesmotic reactions (i and ii), a slightly lower final value (less than 2 kcal/mol away) was obtained (Table 2). All three hyperhomodesmotic ring cleavage reactions proposed for **14** suffer from the Coulomb-term correction limitation and, hence, a moderate inaccuracy, in the range of some 3–4 kcal/mol, must be expected.

Despite these limitations mentioned beforehand, a rough estimation of RSE in the range 36–42 kcal/mol for oxaphosphirenide **14** (Table 2) is enough to conclude that a small decrease in the bond order of the P–C bond (after formal C-deprotonation **1a**→**14**, Table 1) parallels a decrease in the RSE.

## 3. Materials and Methods

### Computational Details

DFT calculations were performed with the ORCA program (version 3.0.3, Mülheim/Ruhr, Germany) [59]. All geometry optimizations were run in redundant internal coordinates in the gas phase, with tight convergence criteria, and using the B3LYP functional [60,61] together with the def2-TZVP basis set [62] and the speeding up algorithm RIJCOSX [63]. The latest Grimme’s semiempirical atom-pair-wise London dispersion correction (DFT-D3) was included in all calculations [64]. Harmonic frequency calculations verified the nature of ground states or transition states (TS) having all real (positive) frequencies or only one imaginary (negative) frequency, respectively. Relaxed force constants were computed at the optimization level and obtained by inversion of the Hessian matrix and appropriate unit conversion of the reciprocal of the resulting diagonal elements. From these optimized geometries all reported data were obtained by means of single-point (SP) calculations using the more polarized def2-TZVPP [65] basis set. Basis sets may be obtained from the Basis Set Exchange (BSE) software and the EMSL Basis Set Library [66,67] Reported energies were corrected for the zero-point vibrational term at the optimization level and obtained by means of the recently developed near linear scaling domain-based local pair natural orbital (DLPNO) method [68] to achieve coupled cluster theory with single-double and perturbative triple excitations (CCSD(T)) [69]. For comparative purposes, local correlation schemes of type LPNO (Local Pair Natural Orbital) for high level single reference methods, such as CEPA (Coupled Electron-Pair Approximation) [70,71], here the slightly modified NCEPA/1 version [72] implemented in ORCA, was used, as well as the spin-component scaled second-order Möller–Plesset perturbation theory (SCS-MP2) level [73,74] and the double-hybrid-meta-GGA functional PWPB95 [75,76], together with the D3 correction (PWPB95-D3) (see the SI). Due to the unavailability of unrestricted formalism for DLPNO/CCSD(T) calculations in the working version of ORCA, the corresponding values in the case of triplet electronic states were taken from the LPNO/NCEPA1 level, making use of the reported small differences between the results in these two levels for closed-shell systems [77]. Figure 2, Figure 3 and Figure 7 were drawn with VMD [78].

## 4. Conclusions

In total, a full picture of the potential energy surfaces (PES) for oxaphosphirene and oxaphosphirenide anion has been presented, including remarkable isomers in the triplet electronic state for the first time. Furthermore, ground state structures of fluorinated derivatives are detailed. The PES of the neutral CHOP system includes important derivatives, such as phosphaketene (phosphaisocyanic acid, H-PCO), linear isomers such as phosphacyanic (H-OCP), and phosphafulminic (H-CPO) acids, as well as two other cyclic isomers and fragmentation products. In the case of the anionic derivatives, the phosphaethynolate anion is the most stable. The electronic structures of the most representative isomers were described using MO theory, NBO analysis, and bond strength descriptors. Using both homodesmotic and hyperhomodesmotic reactions, ring strain energies were estimated for the parent 1*H*-oxaphosphirene, oxaphosphirane-3-ylidene, and the oxaphosphirenide anion, the latter requiring an additional electrostatic energy-correction term.

## Figures and Tables

**Table 1:** Endocyclic bond strength parameters and natural charges.

	Bond	d (Å)	WBI	MBO	ρ (au)	G (au)	G/ρ (au)	Atom	q^nat^ (*e*)
**1a**	P–C P–O C–O	1.671 2.046 1.288	1.571 0.779 1.338	1.694 0.785 1.339	0.183 ^1^ 0.353	0.234 ^1^ 0.463	1.279 ^1^ 1.311	P C O	0.469 −0.137 −0.525
**2a**	P–C P–O C–O	1.956 1.870 1.229	0.997 0.626 1.582	0.991 0.735 1.555	0.116 0.101 0.393	0.044 0.105 0.700	0.376 1.041 1.780	P C O	0.359 0.236 −0.537
**3a*^sp^***	P–C P–O	1.677 1.649	2.107 0.818	1.984 1.036	0.202 0.162	0.254 0.246	1.253 1.521	P C O	0.736 −0.318 −0.909
**3a*^ap^***	P–C P–O	1.664 1.644	2.238 0.819	2.101 1.030	0.204 0.165	0.251 0.251	1.235 1.523	P C O	0.726 −0.311 −0.912
**4a**	P–C P–O C–O	1.672 1.773 1.873	2.005 0.642 0.520	1.917 0.794 0.472	0.198 0.126 ^1^	0.251 0.164 ^1^	1.265 1.306 ^1^	P C O	0.589 −0.302 −0.775
**5a**	P–C P–O	1.562 1.475	2.421 1.401	2.341 1.903	0.206 0.233	0.322 0.518	1.561 2.225	P C O	1.540 −0.867 −0.917
**6a**	P–C C–O	1.683 1.152	1.674 2.022	1.719 2.212	0.163 0.477	0.248 1.014	1.522 2.128	P C O	0.049 0.346 −0.424
**7a**	P–C C–O	1.547 1.299	2.696 1.115	2.833 1.184	0.202 0.333	0.380 0.450	1.878 1.350	P C O	0.327 −0.184 −0.641
**8a**	P–O C–O	1.591 1.249	0.928 1.309	1.197 1.359	0.152 0.328	0.323 0.737	2.125 2.249	P C O	0.421 0.084 −0.639
**9a**	P–O C–O	1.919 1.145	0.410 1.908	0.550 2.079	0.074 0.463	0.077 1.059	1.039 2.289	P C O	0.081 0.538 −0.558
***syn*-6a^t^**	P–C C–O	1.941 1.169	0.851 1.978	0.965 2.202	0.130 1.113	0.043 0.506	0.332 0.454	P C O	0.298 0.223 −0.454
**6a^t^**	P–C C–O	1.963 1.167	0.815 1.991	0.950 2.199	0.125 0.458	0.038 0.952	0.308 2.077	P C O	0.297 0.225 −0.460
**12a^t^**	P–C C–O	1.846 1.212	1.016 1.821	1.142 1.994	0.161 0.418	0.103 0.736	0.640 1.759	P C O	0.339 0.014 −0.473
**14**	P–C P–O C–O	1.820 1.967 1.278	1.471 0.728 1.370	1.572 0.735 1.270	0.146 ^1^ 0.351	0.086 ^1^ 0.537	0.589 ^1^ 1.530	P C O	−0.249 −0.123 −0.628
**15**	P–C C–O	1.618 1.199	2.241 1.638	2.581 1.876	0.178 0.426	0.307 0.768	1.718 1.802	P C O	−0.436 0.088 −0.652
**16**	P–C P–O	1.597 1.519	2.847 1.156	2.727 1.571	0.185 0.211	0.261 0.430	1.414 2.036	P C O	1.146 −1.078 −1.068
**17**	P–O C–O	1.756 1.179	0.647 1.662	0.782 1.784	0.100 0.408	0.168 0.955	1.685 2.338	P C O	−0.528 0.110 −0.583
**15^t^**	P–C C–O	1.792 1.216	1.377 1.639	1.544 1.859	0.152 0.405	0.142 0.735	0.933 1.815	P C O	−0.350 −0.037 −0.613

^1^ No BCP found.

**Table 2:** Computed (CCSD(T)/def2-TZVPP//B3LYP-D3/def2-TZVP) RSE (kcal/mol) using homodesmotic (RC4) or hyperhomodesmotic (RC5) reaction schemes.

	RSE_RC4_	RSE_RC5_
**1a**	49.90	49.08
**2a**	7.53	4.59
**14**	36.83	41.99

## Supplementary Materials

The following are available online, Tables S1 and S2: Computed zero-point corrected relative energies (kcal/mol) at various computational levels, Computed geometries (Cartesian coordinates, in Å), energies (in hartree) and imaginary frequencies (for TS, in cm^−1^) for all computed species.
