# Supplementary material for: Quantum Chemical Calculations on CHOP Derivatives—Spanning the Chemical Space of Phosphinidenes, Phosphaketenes, Oxaphosphirenes, and COP− Isomers
Source: Molecules. 2018 Dec 17;23(12):3341. doi: 10.3390/molecules23123341 (PMC6321265; doi:10.3390/molecules23123341)
Supplement: Supplementary file 1 [file molecules-23-03341-s001.pdf]

Supporting Information

Quantum chemical calculations on CHOP derivatives – spanning the chemical space of phosphinidenes, phosphaketenes, oxaphosphirenes and CPO^- isomers

Alicia Rey, Arturo Espinosa Ferao* and Rainer Streubel*

Table of contents

page

Table S 1: Relative ZPE-corrected relative energies at various computational levels for isomers of **1a** and **14** and their interconversion TSs. S 2

Table S 2: Relative ZPE-corrected relative energies at various computational levels for isomers of **1b-e**. S 3

Calculated structures: Cartesian coordinates and energies for all minima and transition states. S 5

Table S 1. Computed zero-point corrected relative energies (kcal/mol) at various computational levels for isomers of **1a** and **14**.

Level [a)]	**1a**	**(1a-2a)**‡	**(1a-3a**^{sp}**)**‡	**(1a-5a)**‡	**(1a-6a)**‡	**(1a-8a)**‡	**2a**	**(2a-6a)**‡	**(2a-9a)**‡	**3a**^{sp}	**(3a**^{sp}**-4a)**‡	**4a**	**(4a-3a**^{ap}**)**‡	**3a**^{ap}
B3LYP-D3	0.00	44.95	52.63	28.15	12.19	71.48	6.23	16.27	19.45	39.82	41.06	39.79	42.72	41.17
PWBP95-D3	0.00	42.57	50.47	31.44	15.91	76.49	5.52	18.05	20.68	42.02	42.77	39.47	44.88	43.08
SCS-MP2	0.00	42.20	46.68	31.44	13.10	76.97	3.71	15.73	12.78	39.86	41.09	37.80	42.21	41.76
LPNO-NCEPA1	0.00	43.26	48.91	28.94	11.30	70.32	3.15	13.57	10.04	34.59	36.84	36.29	37.42	35.62
DLPNO-CCSD(T)	0.00	42.42	47.73	30.80	13.40	73.61	3.85	15.26	12.72	36.63	38.25	36.33	39.39	37.66

a) With def2-TZVPP basis set, using geometries and ZPE-correction at B3LYP-D3/def2-TZVP.

Table S 1. Continued

Level [a)]	**5a**	**6a**	*l*(**6a-7a**)‡	*b*(**6a-7a**)‡	**7a**	**8a**	**9a**	***syn*-6a**^{t}	**6a**^{t}	**(6a**^{t}**-10a**^{t}**+11)**‡	**(6a**^{t}**-12a**^{t}**)**‡	**12a**^{t}	**10a+11**	**10a**^{t}**+11**
B3LYP-D3	5.87	-47.36	22.84	60.79	-23.27	50.29	16.67	-3.86	-6.10	-6.17	28.00	-7.01	16.56	-16.01
PWBP95-D3	4.59	-46.68	22.04	61.89	-23.89	56.92	19.13	1.00	-1.39	-1.89	32.90	-3.00	17.22	-14.93
SCS-MP2	-2.27	-49.74	17.89	66.06	-29.68	68.00	15.69	0.47	-1.76	-4.02	37.24	0.36	7.47	-24.06
LPNO-NCEPA1	3.32	-46.71	24.67	60.04	-25.38	46.42	14.43	-1.21	-3.64	-6.46	34.33	-6.89	-2.74	-26.07
DLPNO-CCSD(T)	2.35	-46.60	23.17	60.68	-26.04	56.92	16.04	-1.21	-3.64	-6.46	34.33	-6.89	6.19	-26.07

a) With def2-TZVPP basis set, using geometries and ZPE-correction at B3LYP-D3/def2-TZVP.

Table S 1. continuation

Level [a)]	**14**	**15**^{t}	**15**	**(14-16)**‡	**16**	**(14-17)**‡	**17**	*rmsd*
B3LYP-D3	0.00	-21.40	-71.58	15.28	-3.16	30.08	21.66	*4.26*
PWBP95-D3	0.00	-15.87	-70.80	14.77	-5.27	32.51	25.15	*4.27*
SCS-MP2	0.00	-14.61	-75.10	11.94	-12.47	25.10	27.09	*3.64*
LPNO-NCEPA1	0.00	-14.69	-67.78	15.21	-3.09	21.76	25.88	*2.85*
DLPNO-CCSD(T)	0.00	-14.69	-69.34	13.77	-6.33	24.66	27.24	*0.00*

a) With def2-TZVPP basis set, using geometries and ZPE-correction at B3LYP-D3/def2-TZVP.

Table S 2. Computed zero-point corrected relative energies (kcal/mol) at various computational levels for isomers of **1b-e**.

Level [a)]	**1b**	**2b**	**3b^{sp}**	**3b^{ap}**	**4b**	**5b**	**6b**	**7b**	**8b**	**9b**	**10b+11**	***syn*-6b^{t}**	**6b^{t}**	**10b^{t}+11**	**12b^{t}**
B3LYP-D3	0.00	-10.36	128.96	130.26	32.15	25.16	-45.03	54.35	46.86	2.14	-1.00	-21.07	-21.84	-32.19	-15.48
PWBP95-D3	0.00	-12.83	132.16	133.26	30.62	23.86	-45.19	56.85	50.36	2.08	-1.94	-16.78	-18.19	-32.29	-13.65
SCS-MP2	0.00	-14.63	132.71	134.88	33.23	23.44	-48.33	55.92	53.11	-5.74	-11.19	-14.56	-16.95	-40.65	-14.88
LPNO-NCEPA1	0.00	-13.29	125.84	126.75	32.88	22.99	-44.44	56.38	47.24	-5.72	-16.30	-14.41	-16.81	-39.70	-15.63
DLPNO-CCSD(T)	0.00	-13.54	127.34	128.48	33.41	22.96	-44.52	56.30	49.67	-4.39	-10.10	-14.41	-16.81	-39.70	-15.63

a) With def2-TZVPP basis set, using geometries and ZPE-correction at B3LYP-D3/def2-TZVP.

Table S 2. Continued.

Level [a)]	**1c**	**2c**	**3c^{sp}**	**3c^{ap}**	**4c**	**5c**	**6c**	**7c**	**8c**	**9c**	**10c+11**	***syn*-6c^{t}**	**6c^{t}**	**10c^{t}+11**	**12c^{t}**
B3LYP-D3	0.00	10.93	59.76	61.57	58.40	10.58	-36.60	-5.40	51.73	22.57	16.79	0.31	-2.10	-11.54	-4.50
PWBP95-D3	0.00	9.86	61.91	63.55	57.45	10.04	-35.97	-5.77	57.85	24.46	17.03	5.00	2.44	-10.51	-0.71
SCS-MP2	0.00	8.23	59.80	62.47	55.14	5.93	-39.14	-11.94	68.63	19.65	8.90	5.21	2.96	-18.49	0.14
LPNO-NCEPA1	0.00	7.88	54.28	56.01	54.58	9.03	-35.85	-7.42	49.88	18.73	5.13	3.82	1.35	-20.34	-4.57
DLPNO-CCSD(T)	0.00	8.38	56.50	58.29	54.50	8.52	-35.73	-7.91	63.81	20.28	8.69	3.82	1.35	-20.34	-4.57

a) With def2-TZVPP basis set, using geometries and ZPE-correction at B3LYP-D3/def2-TZVP.

Table S 2. Continued.

Level [a)]	**1d**	**2d**	**3d^{sp}**	**3d^{ap}**	**4d**	**5d**	**6d**	**7d**	**8d**	**9d**	**10d+11**	***syn*-6d^{t}**	**6d^{t}**	**10d^{t}+11**	**12d^{t}**
B3LYP-D3	0.00	9.86	41.78	43.93	43.14	7.37	-40.07	-19.10	49.01	16.75	14.94	0.21	-2.87	-14.67	-6.88
PWBP95-D3	0.00	9.28	42.71	45.11	42.91	6.07	-39.24	-20.93	55.59	19.04	15.95	5.44	2.02	-13.00	-3.19
SCS-MP2	0.00	9.06	41.88	43.77	42.50	-0.39	-41.29	-26.81	67.01	16.31	9.07	6.66	3.59	-19.83	1.40
LPNO-NCEPA1	0.00	8.40	35.98	37.21	38.34	6.10	-38.09	-23.07	46.77	15.15	5.64	6.77	3.14	-20.31	-5.13
DLPNO-CCSD(T)	0.00	8.56	37.56	39.08	38.81	4.46	-38.65	-24.05	56.32	15.96	8.47	6.77	3.14	-20.31	-5.13

a) With def2-TZVPP basis set, using geometries and ZPE-correction at B3LYP-D3/def2-TZVP.

Table S 2. Continued.

Level [a)]	**1e**	**2e**	**$3e^{sp}$**	**$3e^{ap}$**	**4e**	**5e**	**6e**	**7e**	**8e**	**9e**	**10e+11**	**$6e^{t}$**	**$10e^{t}$+11**	**$12e^{t}$**	*rmsd*
B3LYP-D3	0.00	11.81	62.93	64.90	61.19	3.79	-36.72	2.58	51.02	14.92	11.28	-9.83	-16.95	-1.75	*4.75*
PWBP95-D3	0.00	10.35	62.25	67.08	61.69	4.77	-36.69	1.68	57.53	15.74	11.74	-8.08	-15.46	1.91	*4.38*
SCS-MP2	0.00	8.39	60.87	66.05	61.24	3.36	-40.22	-4.33	71.55	9.22	4.25	7.98	0.02	30.81	*5.81*
LPNO-NCEPA1	0.00	8.23	59.07	58.39	57.31	4.34	-36.69	-1.15	48.78	6.77	0.13	-10.61	-19.51	3.99	*3.26*
DLPNO-CCSD(T)	0.00	8.54	59.58	61.01	58.18	3.74	-36.99	-1.36	60.36	8.57	3.76	-10.61	-19.51	3.99	*0.00*

a) With def2-TZVPP basis set, using geometries and ZPE-correction at B3LYP-D3/def2-TZVP.

Calculated structures

Cartesian coordinates (in Å), G correction (G-E) and ZPE (in hartrees) for minima and transition states were computed at B3LYP-D3/def2-TZVP. In addition, electronic energies (in hartrees) are quoted at the (gas-phase) DLPNO-CCSD(T)/def2-TZVPP level, unless otherwise stated. Imaginary frequencies are obtained upon frequency calculation (at the optimization level).

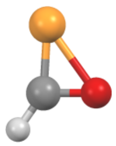

1a:

E = -454.566645531697 au

ZPE = 0.01777141 au

G_{corr} = -0.00629716 au

P	-0.055434	0.325315	-0.372874	O	-0.722582	-0.692650	-2.017852
C	-0.570423	-1.193544	-0.840983	H	-0.775800	-2.225146	-0.566784

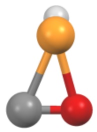

2a:

E = -454.557426929646 au

ZPE = 0.01469222 au

G_{corr} = -0.00937365 au

P	0.128056	0.459021	-0.513575	O	-0.630416	-0.590945	-1.862799
C	-0.770944	-1.248683	-0.834246	H	-1.101207	1.117976	-0.232441

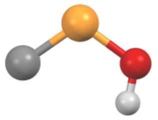

3a^{sp}:

E = -454.50533750778 au

ZPE = 0.01588554 au

G_{corr} = -0.00923382 au

P	0.911959	-0.546979	-1.149077	O	-0.416042	-0.293500	-0.205000
C	2.208721	-0.947946	-0.164304	H	-0.269573	-0.406462	0.744027

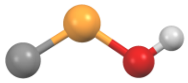

3a^{ap}:

E = -454.50421688159 au

ZPE = 0.01610776 au

G_{corr} = -0.00870501 au

P	0.697515	-0.250573	-1.459120	O	-0.358834	-0.440497	-0.214002
C	-0.025479	0.029813	-2.931086	H	0.114030	-0.601456	0.612272

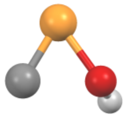

4a:

E = -454.507519853492 au

ZPE = 0.01689948 au

G_{corr} = -0.00752037 au

P	1.112989	0.201462	-1.468573	O	-0.239953	-0.078129	-0.356976
C	-0.255587	-0.423242	-2.197900	H	-0.190217	-0.962805	0.031513

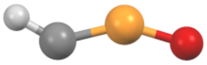

5a:

E = -454.561698777282 au

ZPE = 0.01614212 au

G_{corr} = -0.00750417 au

P	0.212895	-0.009200	-0.219769	O	-0.235482	0.320231	-1.585407
C	-0.094382	-0.177029	1.301974	H	0.443900	-0.455559	2.190270

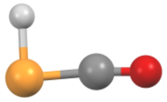

6a:

E = -454.63916619408 au

ZPE = 0.01570129 au

G_{corr} = -0.00803471 au

P	-0.334085	-0.758848	-1.146338	O	-1.756246	-2.157898	-3.156445
C	-1.187498	-1.632808	-2.303602	H	-0.547705	-1.844836	-0.252224

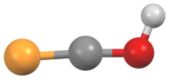

7a:

E = -454.60990385420 au

ZPE = 0.01876157 au

G_{corr} = -0.00453747 au

P	0.905273	0.986458	-1.411710	O	-1.050929	-0.827867	-0.423692
C	-0.131101	-0.015345	-0.850236	H	-0.710057	-1.428582	0.256337

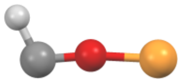

8a:

E = -454.47475080303 au
ZPE = 0.01587422 au
G_{corr} = -0.00748750 au

P	-0.414091	1.021741	-2.619830	O	-0.743722	-0.503719	-2.312679
C	-0.956858	-1.669238	-1.918904	H	-1.323629	-2.310186	-2.739072

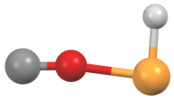

9a:

E = -454.53623239390 au
ZPE = 0.01346790 au
G_{corr} = -0.01115303 au

P	0.128056	0.459021	-0.513575	O	-0.630416	-0.590945	-1.862799
C	-0.770944	-1.248683	-0.834246	H	-1.101207	1.117976	-0.232441

10a:

E = -341.388185592 au
ZPE = 0.00536319 au
G_{corr} = -0.01189675 au

P	0.256820	-0.337953	-0.091519	H	-0.171828	-1.592091	0.438356

11:

E = -113.15769411317 au
ZPE = 0.00504063 au
G_{corr} = -0.01342310 au

C	0.000000	0.000000	0.000950	O	0.000000	0.000000	1.126008

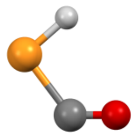

***syn*-6a^{t}:**

E = -454.544514091 au (LPNO-NCEPA1/def2-TZVPP)
ZPE = 0.01306317 au
G_{corr} = -0.01312961 au

P	0.065606	0.402985	0.113929	O	-1.179793	-0.974512	-1.987171
C	-0.364063	-1.006152	-1.150335	H	-0.896262	1.315048	-0.419484

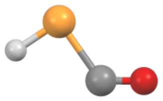

6a^t: E = -454.549224816 au (LPNO-NCEPA1/def2-TZVPP)
ZPE = 0.01364463 au
G_{corr} = -0.01229304 au

P	-0.407365	0.296499	0.211422	O	-0.938249	-1.641475	-1.750903
C	-0.742219	-0.509685	-1.546604	H	-0.286678	1.592029	-0.356975

10a^t: E = -341.424276639186 au (LPNO-NCEPA1/def2-TZVPP)
ZPE = 0.00532479 au
G_{corr} = -0.01297469 au

P	0.257074	-0.337210	-0.091833	H	-0.172082	-1.592835	0.438670

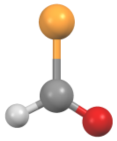

12a^t: E = -454.558753362 au (LPNO-NCEPA1/def2-TZVPP)
ZPE = 0.01736847 au
G_{corr} = -0.00794375 au

P	0.028998	0.336750	-0.036099	O	-0.846811	-0.980857	-2.215640
C	-0.588849	-1.086295	-1.036552	H	-0.717577	-2.055623	-0.510203

TS(**1a**→**2a**): E = -454.49300211132 au
ZPE = 0.01164105 au
G_{corr} = -0.01233572 au
$\nu = -1140.81\ cm^{-1}$

P	0.199044	0.378621	-1.831100	O	0.479417	0.300589	-0.109601
C	0.447967	-0.938405	-0.735971	H	-0.699197	-1.003519	-1.315264

TS(**1a**→**3a**^{sp}): E = -454.48476937244 au
ZPE = 0.01189687 au
G_{corr} = -0.01211720 au
$\nu = -1454.16\ cm^{-1}$

P	0.980392	0.405797	-1.511244	O	0.197397	-0.043218	-0.085466
C	-0.169863	-0.745142	-1.815971	H	-0.580694	-0.880150	-0.579255

TS(**1a**→**5a**):

E = -454.51284816878 au
ZPE = 0.01397131 au
G_{corr} = -0.01101755 au
ν = -466.57 cm^{-1}

P	0.713932	-0.055960	-0.449738	O	-0.590036	0.342094	-1.126198
C	0.228631	-0.355412	1.109131	H	-0.025597	-0.252278	2.153874

TS(**1a**→**6a**):

E = -454.54366044447 au
ZPE = 0.01603645 au
G_{corr} = -0.00792761 au
ν = -790.61 cm^{-1}

P	0.008718	0.249045	-0.024427	O	-0.853212	-0.979029	-2.244476
C	-0.584062	-1.050096	-1.055365	H	-0.695683	-2.005944	-0.474225

TS(**1a**→**8a**):

E = -454.44564603587 au
ZPE = 0.01410887 au
G_{corr} = -0.00999932 au
ν = -612.88 cm^{-1}

P	-0.297597	0.873532	-0.781360	O	-0.296317	-0.675669	-1.448745
C	-0.269236	-1.744713	-0.717177	H	-1.261088	-2.239174	-0.851211

TS(**2a**→**6a**):

E = -454.53732036759 au
ZPE = 0.01318034 au
G_{corr} = -0.01129869 au
ν = -447.86 cm^{-1}

P	0.157369	0.521332	-0.166553	O	-0.963842	-0.733660	-1.896504
C	-0.507530	-1.263507	-0.977771	H	-1.060508	1.213204	-0.402233

TS(**2a**→**9a**):

E = -454.53823445448 au
ZPE = 0.01189951 au
G_{corr} = -0.01443587 au
ν = -185.53 cm^{-1}

P	0.306679	0.955925	-0.319122	O	-0.769347	-0.789193	-1.666229
C	-0.878254	-1.847466	-1.282511	H	-1.033588	1.418103	-0.175198

TS(**3a**^{sp}→**4a**): E = -454.50299327253 au
ZPE = 0.01539002 au
G_{corr} = -0.00900122 au
ν = -239.30 cm^{-1}

P	0.972385	0.255399	-1.461614	O	-0.010354	-0.035499	-0.114651
C	-0.198197	-0.537934	-2.361791	H	-0.336603	-0.944680	-0.053880

TS(**4a**→**3a**^{ap}): E = -454.50123210076 au
ZPE = 0.01546202 au
G_{corr} = -0.00894587 au
ν = -229.01 cm^{-1}

P	0.879357	0.034544	-1.414567	O	-0.283157	-0.186807	-0.232742
C	-0.146020	-0.226752	-2.727937	H	-0.022948	-0.883699	0.383310

TS(**6a**→**7a**)^{lin}: E = -454.52572849952 au
ZPE = 0.01319162 au
G_{corr} = -0.01029514 au
ν = -1758.39 cm^{-1}

P	0.901017	0.703786	-1.150720	O	-1.360018	-0.903147	-0.464369
C	-0.409813	-0.135861	-0.837251	H	-0.118002	-0.950113	0.023040

TS(**6a**→**7a**)^{bent}: E = -454.46350206266 au
ZPE = 0.01131182 au
G_{corr} = -0.01273514 au
ν = -1043.59 cm^{-1}

P	0.302036	0.252952	-0.033445	O	-1.221473	-0.243108	-1.429724
C	-0.365669	-1.106458	-0.935692	H	-1.089405	0.833984	-1.044200

TS(**6a**^{t}→**10a**^{t}+**11**): E = -454.552325432673 au (LPNO-NCEPA1/def2-TZVPP)
ZPE = 0.01252982 au
G_{corr} = -0.01367733 au
ν = -172.78 cm^{-1}

P	-0.376148	0.390065	0.346636	O	-0.956016	-1.707237	-1.797677
C	-0.765901	-0.586755	-1.670992	H	-0.276447	1.641296	-0.321028

TS(**6a^t**→**12a^t**): E = -454.48718046332 au (LPNO-NCEPA1/def2-TZVPP)
ZPE = 0.01130451 au
G_{corr} = -0.01381962 au
ν = -1687.36 cm^{-1}

P	-0.103884	-0.038927	0.061315	O	-1.059761	-1.023764	-2.343561
C	-0.360944	-1.089332	-1.377980	H	-0.599650	-1.634002	-0.138267

1b: E = -553.71324272673 au
ZPE = 0.01083914 au
G_{corr} = -0.01527121 au

P	-0.031824	0.294312	-0.243394	O	-0.759172	-0.741080	-2.117055
C	-0.588491	-1.144614	-0.962986	F	-0.829974	-2.387610	-0.594936

2b: E = -553.73353759587 au
ZPE = 0.00917494 au
G_{corr} = -0.01656146 au

P	0.123343	0.378678	-0.564413	O	-0.597710	-0.598597	-1.875794
C	-0.744022	-1.301780	-0.838790	F	-1.156123	1.259069	-0.164064

3b^{sp}: E = -553.50576212692 au
ZPE = 0.00704385 au
G_{corr} = -0.01982456 au

P	0.947538	-0.549443	-1.252321	O	-0.438408	-0.266858	-0.416302
C	2.157085	-0.936101	-0.137027	F	-0.231148	-0.442485	1.031296

3b^{ap}: E = -553.50340553283 au
ZPE = 0.00690072 au
G_{corr} = -0.02035693 au

P	0.710137	-0.242873	-1.515536	O	-0.481002	-0.412959	-0.376282
C	-0.020966	0.042364	-2.992359	F	0.219062	-0.649246	0.892242

4b: E = -553.65635629915 au
ZPE = 0.00814040 au
G_{corr} = -0.01892253 au

P	0.976783	-0.861146	-1.583123	O	-0.149883	-1.840920	-1.410916
C	-0.037349	0.567564	-1.103811	F	-0.362320	0.871788	0.105914

5b:

E = -553.67548248987 au

ZPE = 0.00950683 au

G_{corr} = -0.01644566 au

P	0.385131	-0.033867	-0.345557	O	-0.357455	0.349352	-1.568093
C	-0.087881	-0.167331	1.223955	F	0.387136	-0.469710	2.376763

6b:

E = -553.78370365372 au

ZPE = 0.01050660 au

G_{corr} = -0.01575798 au

P	-0.355564	-0.760627	-1.203272	O	-1.784941	-2.140072	-3.260094
C	-1.221172	-1.637711	-2.389310	F	-0.463857	-1.855982	-0.005933

7b:

E = -553.62135694967 au

ZPE = 0.00911392 au

G_{corr} = -0.01744199 au

P	0.985208	1.014187	-1.409168	O	-1.140742	-0.630199	-0.639638
C	-0.204242	0.057943	-0.944008	F	-0.627038	-1.727266	0.563514

8b:

E = -553.63205829865 au

ZPE = 0.00909438 au

G_{corr} = -0.01730042 au

P	-0.008672	-0.000049	-0.000008	O	1.589087	0.112593	0.000072
C	2.865149	-0.015251	-0.000042	F	3.391326	1.211650	0.003113

9b:

E = -553.71317136731 au

ZPE = 0.00763393 au

G_{corr} = -0.02234184 au

P	0.372670	1.121589	-0.382689	O	-0.536564	-1.053763	-1.286600
C	-1.085408	-1.973583	-1.642297	F	-1.125210	1.643126	-0.131476

10b:

E = -440.554933616448 au

ZPE = 0.00696305 au

G_{corr} = -0.03197566 au

P	0.282750	-0.262088	-0.123572	F	-0.197758	-1.667956	0.470409

***syn*-6b^t^:** E = -553.71176506367 au (LPNO-NCEPA1/def2-TZVPP)
ZPE = 0.00908029 au
G_{corr} = -0.01871518 au

P	0.026760	0.301502	0.008229	O	-1.100639	-1.267058	-2.071706
C	-0.427517	-0.934871	-1.168475	F	-0.873116	1.637796	-0.211109

6b^t^: E = -553.71558270031 au (LPNO-NCEPA1/def2-TZVPP)
ZPE = 0.00889092 au
G_{corr} = -0.01872238 au

P	-0.422124	0.228443	0.197696	O	-0.945280	-1.660460	-1.797635
C	-0.741152	-0.548960	-1.486222	F	-0.265956	1.718346	-0.356899

10b^t^: E = -440.59115049921 au (LPNO-NCEPA1/def2-TZVPP)
ZPE = 0.00189888 au
G_{corr} = -0.01961945 au

P	0.283416	-0.260140	-0.124396	F	-0.198424	-1.669905	0.471232

12b^t^: E = -553.71616085576 au (LPNO-NCEPA1/def2-TZVPP)
ZPE = 0.01085667 au
G_{corr} = -0.01625225 au

P	0.035949	0.355848	-0.028466	O	-0.847031	-0.947559	-2.253251
C	-0.584056	-1.009976	-1.098995	F	-0.729101	-2.184337	-0.417782

1c: E = -493.82159926408 au
ZPE = 0.04583705 au
G_{corr} = 0.01909055 au

P	-0.066479	0.313133	-0.388336	H	-1.899193	-2.852061	-0.661078
C	-0.573634	-1.225267	-0.820968	H	-0.237902	-3.289018	-1.059708
O	-0.721038	-0.719018	-2.002498	H	-0.645848	-2.800490	0.607191
C	-0.851754	-2.623949	-0.446526				

2c: E = -493.80579347409 au
ZPE = 0.04347979 au
G_{corr} = 0.01663877 au

P	0.016432	0.113298	-0.048662	H	0.747433	-0.633053	-2.307151
C	1.933175	-0.082800	0.135824	H	-1.015606	-0.547916	-2.110279
O	1.585599	1.102948	0.047298	H	-0.093311	0.936454	-2.366360
C	-0.070104	-0.044583	-1.888987				

3c^{sp}:

E = -493.72981449004 au
ZPE = 0.04472058 au
G_{corr} = 0.01734763 au

P	0.972504	-0.545798	-1.295042	H	-1.269154	-0.216652	1.439810
C	2.327505	-0.949512	-0.395189	H	0.014262	-1.453083	1.317073
O	-0.347684	-0.297257	-0.377525	H	0.448513	0.274530	1.460476
C	-0.275949	-0.433970	1.053702				

3c^{ap}:

E = -493.72556259032 au
ZPE = 0.04436594 au
G_{corr} = 0.01594641 au

P	0.650722	-0.220591	-1.657276	H	-0.604387	-0.756810	1.597893
C	-0.085699	0.071024	-3.117970	H	0.821631	-1.571056	0.907631
O	-0.349906	-0.410619	-0.382047	H	0.857153	0.188246	1.219243
C	0.232762	-0.652155	0.911601				

4c:

E = -493.73400691678 au
ZPE = 0.04454938 au
G_{corr} = 0.01834825 au

P	-0.016799	-0.065727	0.065753	H	-0.054537	2.103368	-1.892309
C	1.653408	0.031410	0.121604	H	1.261142	3.125150	-1.229629
O	0.761650	1.529658	-0.066950	H	1.636575	1.532523	-1.956968
C	0.910541	2.105609	-1.382835				

5c:

E = -493.80640281061 au
ZPE = 0.04466565 au
G_{corr} = 0.01747604 au

P	0.279657	-0.003815	-0.305371	H	-0.332776	-0.429556	3.256517
C	0.005714	-0.203760	1.232915	H	0.867535	-1.561858	2.645470
O	-0.355129	0.356887	-1.597146	H	1.289203	0.153069	2.907175
C	0.511796	-0.532650	2.563559				

6c:

E = -493.87745290795 au
ZPE = 0.04560641 au
G_{corr} = 0.01801054 au

P	-0.391118	-0.716806	-1.238163	H	-0.031842	-2.953923	-0.153169
C	-1.201585	-1.605668	-2.406934	H	0.060189	-1.588893	0.965653
O	-1.745406	-2.146091	-3.271912	H	-1.524221	-2.183170	0.451708
C	-0.498474	-2.013681	0.131565				

7c: E = -493.83609278851 au
ZPE = 0.04766377 au
G_{corr} = 0.02097882 au

P	0.954673	1.125974	-1.510521	H	-1.613386	-2.165637	0.620701
C	-0.143742	0.153510	-1.009013	H	0.078798	-2.247807	0.035770
O	-1.073643	-0.639425	-0.613072	H	-0.373534	-1.064954	1.301425
C	-0.710799	-1.598685	0.413382				

8c: E = -493.71782331623 au
ZPE = 0.04462503 au
G_{corr} = 0.01699575 au

P	0.046181	-0.030580	0.347033	H	4.560494	1.300286	-0.223786
C	2.797476	0.087936	-0.351726	H	3.073477	2.166069	-0.678630
O	1.595300	0.055753	-0.038626	H	3.307953	1.658047	1.003725
C	3.492445	1.376081	-0.041204				

9c: E = -493.78333379081 au
ZPE = 0.04219441 au
G_{corr} = 0.01313817 au

P	0.069153	0.131476	-0.475592	H	-1.007842	2.173109	-0.338521
C	3.131630	-0.246013	0.418225	H	0.139984	2.159126	0.996394
O	2.051776	-0.044856	0.118815	H	0.678547	2.555008	-0.669851
C	0.028594	1.922426	-0.063113				

10c: E = -380.62935904014 au
ZPE = 0.03439641 au
G_{corr} = 0.01181850 au

P	-0.075213	-0.438632	2.080685	H	-0.211541	-1.679635	-0.092097
C	0.400064	-1.629917	0.810989	H	0.723236	-2.619508	1.139691
H	1.330403	-1.086081	0.518805				

***syn*-6c^t:** E = -493.78782380209 au (LPNO-NCEPA1/def2-TZVPP)
ZPE = 0.04282448 au
G_{corr} = 0.01359239 au

P	0.053288	0.401134	0.042998	H	-2.195804	1.198664	-0.542097
C	-0.345100	-1.052857	-1.132363	H	-1.150828	2.520306	0.036427
O	-1.168052	-1.080696	-1.971004	H	-0.975906	1.885203	-1.616029
C	-1.189127	1.620185	-0.577673				

6c^t: E = -493.79222589514 au (LPNO-NCEPA1/def2-TZVPP)
ZPE = 0.04304769 au
G_{corr} = 0.01406857 au

P	0.053681	-0.035819	0.000349	H	-1.117009	-2.164390	0.008865
C	1.980951	0.045099	0.001568	H	0.423127	-2.293424	-0.871893
O	2.649387	1.006647	-0.001896	H	0.421564	-2.285484	0.893434
C	-0.067338	-1.871419	0.008455				

10c^t: E = -380.66468469400 au (LPNO-NCEPA1/def2-TZVPP)
ZPE = 0.03555530 au
G_{corr} = 0.01195761 au

P	-0.185656	-0.487599	2.128461	H	-0.230226	-1.653749	-0.032187
C	0.483074	-1.570942	0.792560	H	0.682062	-2.576486	1.173367
H	1.417696	-1.164999	0.395871				

12c^t: E = -493.80508678852 au (LPNO-NCEPA1/def2-TZVPP)
ZPE = 0.04567013 au
G_{corr} = 0.01749059 au

P	-0.021577	0.114433	-0.000068	H	2.119361	-1.986928	0.879009
C	1.836296	-0.054579	0.000102	H	2.119142	-1.987240	-0.878241
O	2.492885	0.966372	0.000129	H	3.541214	-1.352469	0.000104
C	2.454134	-1.429839	0.000242				

1d: E = -791.23258653958 au
ZPE = 0.02303539 au
G_{corr} = -0.00817368 au

P	-0.057005	0.294168	-0.337218	F	-0.594267	-2.810978	0.838938
C	-0.576432	-1.199958	-0.856076	F	-2.142764	-2.936177	-0.682246
O	-0.725944	-0.708283	-2.031047	F	-0.094781	-3.471534	-1.171864
C	-0.857557	-2.629640	-0.461780				

2d: E = -791.21841893378 au
ZPE = 0.02086143 au
G_{corr} = -0.00870041 au

P	-0.019669	0.027770	0.057475	F	0.775366	-0.649310	-2.497312
C	1.942980	-0.021505	0.024582	F	-1.342609	-0.846467	-2.020738
O	1.477596	1.116394	-0.018623	F	-0.471996	1.113183	-2.402091
C	-0.243624	-0.081693	-1.828156				

3d^{sp}:

E = -791.17127633892 au

ZPE = 0.02158515 au

G_{corr} = -0.00962331 au

P	1.052985	-0.412378	-1.297875	F	-1.478338	-0.563934	1.440151
C	2.020217	-1.538667	-0.495569	F	0.268190	0.670762	1.764025
O	-0.230995	-0.041984	-0.282427	F	0.511470	-1.435482	1.308461
C	-0.234131	-0.333446	1.041732				

3d^{ap}:

E = -791.16902929464 au

ZPE = 0.02117590 au

G_{corr} = -0.00945283 au

P	0.791733	-0.160248	-1.664432	F	-0.807125	-0.771294	1.688475
C	-0.242779	-0.011901	-2.981635	F	1.012479	0.347879	1.305850
O	-0.265984	-0.384773	-0.379936	F	0.944999	-1.773890	0.890021
C	0.222480	-0.642803	0.862682				

4d:

E = -791.17048683714 au

ZPE = 0.02134604 au

G_{corr} = -0.00841900 au

P	0.027482	-0.021112	0.059081	F	-0.270887	2.125297	-2.049671
C	1.685295	-0.164928	0.292327	F	1.311346	3.369697	-1.236681
O	0.748467	1.572730	-0.139561	F	1.784470	1.457578	-2.136866
C	0.900645	2.116563	-1.391459				

5d:

E = -791.22431084319 au

ZPE = 0.02240787 au

G_{corr} = -0.00934198 au

P	0.198337	0.005829	-0.251237	F	-0.462096	-0.439612	3.507098
C	-0.110214	-0.176534	1.258492	F	1.491922	0.299823	2.914061
O	-0.220630	0.328350	-1.623748	F	0.981908	-1.779160	2.591109
C	0.490442	-0.526372	2.558935				

6d:

E = -791.29550909660 au

ZPE = 0.02283200 au

G_{corr} = -0.00684479 au

P	-0.381414	-0.644970	-1.247291	F	-1.766392	-2.248895	0.502382
C	-1.185670	-1.607216	-2.377850	F	0.036374	-3.178525	-0.245759
O	-1.718356	-2.183677	-3.215584	F	0.171942	-1.536084	1.168900
C	-0.507587	-1.997441	0.100084				

7d: E = -791.27286691767 au

ZPE = 0.02443520 au

G_{corr} = -0.00622710 au

P	0.916720	1.152505	-1.549204	F	-1.797979	-2.286528	0.665392
C	-0.122495	0.155792	-1.003336	F	-0.293131	-0.977940	1.512796
O	-1.049580	-0.666666	-0.579144	F	0.255202	-2.405419	-0.016175
C	-0.706124	-1.588684	0.405657				

8d: E = -791.14386595240 au

ZPE = 0.02247316 au

G_{corr} = -0.00714421 au

P	0.023557	0.023691	0.139247	F	3.558215	1.756350	1.260818
C	2.837649	-0.007044	-0.128517	F	4.715453	1.345279	-0.531765
O	1.595277	0.056766	0.001055	F	2.782372	2.319099	-0.692804
C	3.481597	1.372054	-0.030597				

9d: E = -791.20317338993 au

ZPE = 0.02017641 au

G_{corr} = -0.01215414 au

P	0.088377	0.061631	-0.543474	F	-1.155869	2.304822	-0.516859
C	3.003262	-0.135319	0.506753	F	0.170448	2.178461	1.215171
O	1.928924	-0.015190	0.139860	F	0.994279	2.652695	-0.714422
C	0.066292	1.883149	-0.101118				

10d: E = -678.04157950989 au

ZPE = 0.01324336 au

G_{corr} = -0.01455814 au

P	0.085118	-0.015635	-0.154266	F	0.563148	-2.158557	-1.068616
C	-0.110993	-1.831845	0.103053	F	0.521747	-2.434414	1.119802
F	-1.330199	-2.385292	0.040181				

***syn*-6d^{t}:** E = -791.18005675158 au (LPNO-NCEPA1/def2-TZVPP)

ZPE = 0.02030920 au

G_{corr} = -0.01300664 au

P	0.065405	0.363198	0.065906	F	-2.467090	1.177370	-0.495984
C	-0.326160	-1.091505	-1.140385	F	-1.134183	2.765260	0.149761
O	-1.119119	-1.146034	-1.995262	F	-0.973170	1.968861	-1.864831
C	-1.205702	1.633520	-0.583477				

6d^t: E = -791.18619523734 au (LPNO-NCEPA1/def2-TZVPP)
ZPE = 0.02053755 au
G_{corr} = -0.01265955 au

P	0.005363	0.030679	-0.000590	F	-1.337010	-2.274111	0.010111
C	1.983231	0.040208	0.001712	F	0.539152	-2.402761	-1.075061
O	2.658186	0.986467	-0.000969	F	0.538948	-2.392203	1.096727
C	-0.052105	-1.866934	0.008224				

10d^t: E = -678.06140975210 au (LPNO-NCEPA1/def2-TZVPP)
ZPE = 0.01369531 au
G_{corr} = -0.01482099 au

P	0.001312	0.046817	-0.000066	F	0.555570	-2.367133	-1.075037
C	-0.056893	-1.844578	0.008312	F	0.554618	-2.356936	1.097022
F	-1.325786	-2.303913	0.009923				

12d^t: E = -791.20450838549 au (LPNO-NCEPA1/def2-TZVPP)
ZPE = 0.02303582 au
G_{corr} = -0.00752950 au

P	-0.031449	0.097449	0.000003	F	2.055237	-2.114457	1.088681
C	1.813814	-0.015328	0.000063	F	2.055151	-2.114837	-1.087852
O	2.512490	0.967716	-0.000061	F	3.785283	-1.378338	0.000216
C	2.458278	-1.431060	0.000278				

1e: E = -1180.83124894528 au
ZPE = 0.05876638 au
G_{corr} = 0.02078609 au

P	-0.069631	0.270522	-0.371373	C	-1.634496	-4.834734	-1.112000
C	-0.591930	-1.227015	-0.895086	C	-1.359015	-3.528373	-1.484160
O	-0.712213	-0.626745	-2.046383	F	-0.217567	-2.177781	1.674320
C	-0.877171	-2.594664	-0.556249	F	-0.756955	-4.724200	2.403493
C	-0.678861	-3.032094	0.760744	F	-1.688865	-6.484265	0.562488
C	-0.948729	-4.330792	1.146137	F	-2.096263	-5.709407	-2.003332
C	-1.428460	-5.234743	0.202807	F	-1.564568	-3.180995	-2.748446

2e: E = -1180.81667738671 au
ZPE = 0.05673678 au
G_{corr} = 0.01981608 au

P	-0.062877	0.127430	-0.020523	C	-0.209659	-0.013156	-1.842929
C	1.863073	-0.236613	0.113846	C	0.156059	-1.166916	-2.541698
O	1.625802	0.965646	0.045778	C	-0.048095	-1.295544	-3.906661

C	-0.640523	-0.252576	-4.608020	F	0.308995	-2.406290	-4.550633
C	-1.021343	0.908398	-3.946775	F	-0.846390	-0.366619	-5.914014
C	-0.803686	1.012293	-2.582638	F	-1.588028	1.904002	-4.627920
F	0.718690	-2.192140	-1.901654	F	-1.176083	2.145042	-1.981960

3e*sp*:

E = -1180.73483724830 au

ZPE = 0.05702998 au

G_{corr} = 0.01931626 au

P	0.924075	-0.538797	-1.287798	C	0.375666	-1.987391	2.831148
C	1.954536	-0.900707	0.029575	C	-0.061221	-1.775563	1.536928
O	-0.446339	-0.274490	-0.326678	F	0.601053	1.776407	1.200948
C	0.053134	-0.504026	0.938009	F	1.487496	1.345796	3.728395
C	0.551827	0.560159	1.717497	F	1.332502	-1.137752	4.800875
C	0.987428	0.343243	3.011897	F	0.295720	-3.197011	3.378208
C	0.900374	-0.928846	3.565514	F	-0.589891	-2.773155	0.847976

3e*ap*:

E = -1180.73154036521 au

ZPE = 0.05633274 au

G_{corr} = 0.01829737 au

P	0.764454	-0.281984	-1.567789	C	0.345843	-1.997057	2.938500
C	-0.088134	0.079069	-2.963106	C	-0.010012	-1.847610	1.605162
O	-0.324992	-0.459994	-0.313045	F	0.764644	1.645373	1.093812
C	0.117184	-0.614202	0.971970	F	1.473112	1.357636	3.702962
C	0.627699	0.461724	1.693396	F	1.191839	-1.058297	4.924964
C	0.993105	0.318263	3.023752	F	0.211138	-3.177107	3.539079
C	0.848578	-0.914075	3.647827	F	-0.480196	-2.893811	0.928948

4e:

E = -1180.73601499015 au

ZPE = 0.05676375 au

G_{corr} = 0.01826329 au

P	-0.213747	0.288294	0.377582	C	2.468324	2.661232	-3.225796
C	1.295656	-0.366401	0.072178	C	2.320679	2.147643	-1.945563
O	0.952067	1.556187	-0.096359	F	-1.280118	2.283430	-1.596019
C	1.053328	2.009271	-1.379013	F	-0.990491	3.275950	-4.103175
C	-0.056955	2.398396	-2.124748	F	1.495078	3.538452	-5.183565
C	0.083675	2.906739	-3.406995	F	3.681835	2.792430	-3.757424
C	1.350499	3.040286	-3.958299	F	3.397876	1.788809	-1.258266

5e:

E = -1180.82389173824 au

ZPE = 0.05832563 au

G_{corr} = 0.01938551 au

P	0.342250	-0.018064	-0.359213	C	-0.042508	-0.166065	1.183340

O	-0.354535	0.352244	-1.614278	C	0.292801	-1.811138	2.973846
C	0.381787	-0.489719	2.477415	F	0.964761	1.752745	2.964233
C	0.849830	0.499024	3.374115	F	1.646589	1.134403	5.505199
C	1.203627	0.194358	4.673554	F	1.441377	-1.418887	6.361742
C	1.100279	-1.121024	5.119045	F	0.553586	-3.374991	4.724320
C	0.643041	-2.126900	4.271263	F	-0.128819	-2.780463	2.176824

6e:

E = -1180.89147106483 au

ZPE = 0.05866086 au

G_{corr} = 0.02206112 au

P	-0.374366	-0.771893	-1.234581	C	-0.766358	-4.090490	1.282898
C	-1.230913	-1.672746	-2.384086	C	-0.790891	-3.295322	0.148741
O	-1.783010	-2.103394	-3.295311	F	0.296546	-0.143382	1.510628
C	-0.443101	-1.946416	0.175495	F	0.363341	-1.675976	3.726366
C	-0.055378	-1.434052	1.415026	F	-0.348548	-4.302056	3.592812
C	-0.015281	-2.207160	2.563979	F	-1.111917	-5.375983	1.213002
C	-0.375980	-3.545659	2.498741	F	-1.166973	-3.869958	-1.002591

7e:

E = -1180.83565400518 au

ZPE = 0.05955003 au

G_{corr} = 0.02301886 au

P	0.931863	1.034438	-1.448027	C	-0.654850	-3.997811	1.198635
C	-0.118532	0.046281	-0.900072	C	-0.913978	-3.042041	0.225760
O	-1.045740	-0.768814	-0.478331	F	-0.110435	-0.015545	2.041150
C	-0.720278	-1.694286	0.499685	F	0.433456	-1.882045	3.927837
C	-0.270873	-1.304762	1.756205	F	0.050132	-4.519188	3.383356
C	-0.000557	-2.258722	2.727110	F	-0.838159	-5.288829	0.931022
C	-0.196749	-3.605220	2.449158	F	-1.348476	-3.425447	-0.971564

8e:

E = -1180.73572831149 au

ZPE = 0.05808658 au

G_{corr} = 0.02144923 au

P	0.769306	-0.927071	1.316982	C	4.680511	3.284423	-1.213184
C	2.282541	0.503469	-0.597449	C	3.828655	2.208150	-1.386137
O	1.651103	-0.069436	0.324059	F	2.766564	1.675290	2.042079
C	3.145268	1.613790	-0.305466	F	4.425400	3.757632	2.368751
C	3.378445	2.176661	0.966410	F	5.688159	4.848794	0.229432
C	4.227493	3.250286	1.151930	F	5.312500	3.824559	-2.254708
C	4.879319	3.810023	0.058006	F	3.662748	1.736920	-2.616801

9e:

E = -1180.81198591314 au

ZPE = 0.05515847 au

G_{corr} = 0.01517864 au

P	0.030491	-0.111375	-0.336800	C	4.075023	0.453053	-0.611772
C	-0.271417	-0.086296	3.570704	C	2.706550	0.626472	-0.693871
O	-0.164992	0.274965	2.509157	F	1.584732	-2.375846	1.010301
C	1.785937	-0.317457	-0.171309	F	4.238665	-2.667151	1.231862
C	2.359311	-1.440602	0.476938	F	5.888775	-0.833253	0.139371
C	3.723424	-1.609843	0.603444	F	4.921259	1.345673	-1.127356
C	4.580223	-0.665245	0.044185	F	2.275667	1.722053	-1.302427

10e:

E = -1067.64779917582 au

ZPE = 0.04972881 au

G_{corr} = 0.01444629 au

P	-0.374229	-0.771914	-1.234592	C	-0.790847	-3.295309	0.148719
C	-0.443052	-1.946398	0.175478	F	0.296549	-0.143371	1.510607
C	-0.055375	-1.434050	1.415017	F	0.363283	-1.675989	3.726375
C	-0.015303	-2.207151	2.563967	F	-0.348609	-4.302033	3.592816
C	-0.376010	-3.545651	2.498728	F	-1.111894	-5.375978	1.212943
C	-0.766341	-4.090485	1.282875	F	-1.166903	-3.869994	-1.002603

6e^t:

E = -1180.76800126197 au (LPNO-NCEPA1/def2-TZVPP)

ZPE = 0.05505857 au

G_{corr} = 0.01435574 au

P	-0.606048	-0.060074	-0.655732	C	-0.264285	-4.105241	-1.224888
C	2.644184	-0.233511	0.811131	C	-0.451560	-2.750075	-1.425434
O	3.013334	0.688921	1.338609	F	-0.023305	-1.504948	1.940287
C	-0.379582	-1.819566	-0.378541	F	0.349321	-4.145901	2.346215
C	-0.105897	-2.336543	0.895738	F	0.195158	-5.873965	0.260561
C	0.088837	-3.685637	1.122189	F	-0.342743	-4.966767	-2.239331
C	0.009538	-4.574834	0.055486	F	-0.706080	-2.325690	-2.667912

10e^t:

E = -1067.62126764170 au (LPNO-NCEPA1/def2-TZVPP)

ZPE = 0.04952325 au

G_{corr} = 0.01344150 au

P	-0.440427	-0.896790	-1.289145	C	-0.780490	-3.278591	0.146469
C	-0.419024	-1.923633	0.185583	F	0.313940	-0.132189	1.549656
C	-0.038259	-1.417314	1.437629	F	0.357717	-1.690444	3.751932
C	-0.011996	-2.200633	2.576766	F	-0.358118	-4.303180	3.580179
C	-0.377388	-3.540315	2.492913	F	-1.113269	-5.366676	1.200943
C	-0.764150	-4.082095	1.271318	F	-1.157266	-3.826463	-1.013914

12e^t:

E = -1180.74969417509 au (LPNO-NCEPA1/def2-TZVPP)

ZPE = 0.05872858 au

G_{corr} = 0.01931065 au

P	-0.899409	0.265075	0.022931	C	-1.726134	-4.786920	-1.088928
C	-0.500847	-1.187799	-1.049522	C	-1.416598	-3.493568	-1.480954
O	-0.017388	-0.982711	-2.145547	F	-0.001599	-2.200003	1.586393
C	-0.836662	-2.580283	-0.597163	F	-0.585245	-4.715309	2.345734
C	-0.577528	-3.021115	0.699213	F	-1.732081	-6.435399	0.590224
C	-0.865400	-4.312715	1.107455	F	-2.292861	-5.637108	-1.943061
C	-1.445344	-5.197138	0.207839	F	-1.727628	-3.130293	-2.721653

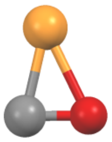

14:

E = -453.97856091269 au

ZPE = 0.00536623 au

G_{corr} = -0.01853277 au

P	-0.050138	0.380955	-0.411825	O	-0.696381	-0.647570	-1.958824
C	-0.601921	-1.294263	-0.861060				

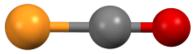

15:

E = -454.09833142571 au

ZPE = 0.00724318 au

G_{corr} = -0.00926679 au

P	-0.350567	-0.786646	-1.152065	O	-1.764723	-2.177558	-3.152940
C	-1.162539	-1.585351	-2.301380				

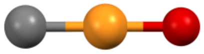

16:

E = -453.99263504073 au

ZPE = 0.00534440 au

G_{corr} = -0.01455094 au

P	-0.042542	0.046293	-0.193798	O	-0.107564	0.303877	-1.689034
C	0.033137	-0.216168	1.379630				

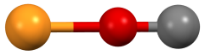

17:

E = -453.94294233407 au

ZPE = 0.00576463 au

G_{corr} = -0.01074478 au

P	0.177418	0.793711	-0.518958	O	-0.512517	-0.701135	-1.129678
C	-0.975569	-1.704951	-1.539408				

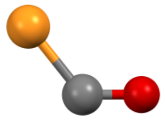

15[t]: E = -453.98462568935 au (LPNO-NCEPA1/def2-TZVPP)

ZPE = 0.00605288 au

G_{corr} = -0.01879663 au

P	0.050376	0.464667	-0.087814	O	-0.853189	-1.057706	-2.158600
C	-0.545625	-0.967840	-0.985296				

TS(**14**→**16**): E = -453.95566135895 au

ZPE = 0.00429672 au

G_{corr} = -0.01949107 au

ν = -140.73 cm^{-1}

P	-0.156749	0.181181	-0.634119	O	-0.642742	-0.311609	-2.104474
C	-0.548949	-1.430451	-0.493117				

TS(**14**→**17**): E = -453.93764837252 au

ZPE = 0.00494610 au

G_{corr} = -0.02014762 au

ν = -417.82 cm^{-1}

P	0.214885	1.217401	-0.230871	O	-0.677163	-0.855058	-1.652460
C	-0.886161	-1.923222	-1.348378				
